# Newly Emerging Parasitic Threats for Human Health: National and International Trends

**DOI:** 10.1155/2016/4283270

**Published:** 2016-02-04

**Authors:** Lidia Chomicz, David Bruce Conn, Jacek P. Szaflik, Beata Szostakowska

**Affiliations:** ^1^Department of Medical Biology, Medical University of Warsaw, 73 Nowogrodzka Street, 02-018 Warsaw, Poland; ^2^Department of Invertebrate Zoology, Museum of Comparative Zoology, Harvard University, Cambridge, MA 02138, USA; ^3^One Health Center, Berry College, School of Mathematical and Natural Sciences, Mount Berry, GA 30149-5036, USA; ^4^Department of Ophthalmology, SPKSO Ophthalmic Hospital, Medical University of Warsaw, 13 Sierakowskiego Street, 03-709 Warsaw, Poland; ^5^Department of Tropical Parasitology, Institute of Maritime and Tropical Medicine, The Medical University of Gdańsk, 9b Powstania Styczniowego Street, 81-519 Gdynia, Poland

Parasites impair healthy life and development in hundreds of millions of individuals throughout the world. Various human populations are at serious risk of illness and even death from these pathogens. It should be taken into account that medically important parasites are not only those often infecting human hosts but also species that rarely colonize the human organism in the given region of the world. The latter can induce serious diseases or high mortality rates as they may be not correctly diagnosed or may be left untreated (e.g., dirofilariosis and malaria in Poland). Recently, reemerging parasites and newly emerging species (e.g., free living, facultative parasitic amoebae) have become an increasing worldwide public health problem connected with many mistakes and uncertainties in diagnosis; thus, there is a need for introduction of new diagnostic methods and successive advances in therapeutic management.

The available evidence indicates that there are changes in natural and anthropogenic environments that affect the spread and emergence of these diseases in any given area, including climate change, population growth agricultural intensification, and human encroachment into wildlife habitats. Likewise, increasing environmental pollution and degradation and alterations in microbial and other ecosystems influence the spread, prevalence, and epidemiological dynamics of parasitic infections. This issue addresses the region-dependent, recurrent, and newly emerging health threats generated by the parasites and related pathogens in specific countries. Yet, while the focus of each article in this issue is on specific geopolitical entities, each of these national or regional cases provides a model that can be applied globally to parasitic threats to any country or region. Among different causes, these threats are due to rising numbers of people traveling greater distances, who undertake trips to endemic countries [1].

For example, dengue fever, an emerging vector-borne disease, currently is threatening human populations in different countries of Asia, the Pacific, the Americas, Africa, and Caribbean, some of which are the popular tourist destinations. The World Health Organization and Centers for Disease Control and Prevention reported that the pathogens occur in about 100 countries. Causative agent of the disease is RNA DENV that shows unexpected geographical expansion and an increase in number and severity of outbreaks in the last decades. Dengue infections are transmitted to vertebrate hosts by the external blood-sucking vectors, usually* Aedes* mosquitoes. The geographical range of dengue incidences is expanding; they are much more widespread than has been suggested previously. Climate changes and the human movement between population centers are, among others, important factors of dengue spread. The available data on spread of the viral disease using a novel approach indicates that dengue incidences occur in 128 countries including those previously classified as dengue-free [2]. At present, dengue is one of the most important mosquito-borne viral diseases in the world that additionally may be transmitted from an infected pregnant mother to her fetus. Although* Aedes aegypti*, the main vector of DENV feeding on human blood, do not remain alive in the winter in temperate zones, not only can some* Aedes* species be active throughout the year in tropical and subtropical zones but they remain alive throughout the winter in the egg stage in temperate climates. This may include invasive species such as* Aedes albopictus,* which has recently colonized vast temperate areas of Europe and North America [3], where it threatens broader transmission of dengue and other diseases such as emerging human dirofilariosis across many new regions of Europe [4]. No diagnostic tests were performed in Poland regarding dengue virus infections until 2005, although, in Europe, sporadic indigenous dengue transmission cases have been detected. The number of Polish travelers to subtropics and tropics, including endemic regions in which dengue fever occurs, increases every year. There was a need to examine and assess a risk of dengue infection during a travel as well as the imported dengue cases. The serological tests performed in the University Centre for Maritime and Tropical Medicine allowed diagnosing the dengue in 149 travelers (19.8% of the investigated group) and indicated a serious risk of dengue virus infection during stays in endemic regions [5].

While these high-profile pandemic and emerging diseases often gain considerable attention from researchers and the popular press, national health ministries, hospitals, and regionally affiliated universities must focus on the health situations and threats that are encountered in their specific areas. Many of these threats come from major neglected diseases that have posed threats to human health for centuries but may be emerging in certain regions or may be emerging within specific groups of patients (e.g., pregnant women), under specific healthcare contexts (e.g., as sequelae to previously treated or cured infections), or under newly emerging situations (e.g., emerging drug resistance). In this issue, some of the most devastating and widespread diseases are presented as they relate to these particular contexts in specific regions of concern. Examples of current health threats in diverse regions and socioeconomic contexts in Asia, North America, South America, and Europe, as well as threats from global migration, are included (e.g., cutaneous leishmaniosis, malaria, Chagas disease, echinococcosis (hydatid disease), and amoebic keratitis). Thus, the issue presents a broad view portraying the intersection of national, regional, and global health. Malaria is among the most prevalent diseases across the globe; its impact on a particular semiurban community in India and within a particular group—pregnant women—is presented; it thus addresses the global public health priority of maternal and child health. Leishmaniosis, which is endemic in many areas, is reported from a nonendemic area in Brazil; the impact of the health-seeking behavior is examined among patients with post-Kala-azar dermal leishmaniosis in Bangladesh.

Besides diverse geographical and biotic situations that affect human health differently in various regions, specific healthcare practices in particular regions may impact the emergence or maintenance of infectious disease. The effective healthcare needs to establish criteria for designating a disease as cured following therapy; methods applied in Brazil and elsewhere and questions of whether it is time to adopt new concepts in dealing with posttherapeutic cure criterion for Chagas disease are critically examined. As a major disease endemic in Brazil and broadly throughout South and Central America, but experiencing a major emergence in both North America and southwestern Europe, this provides an example of how policy in an experienced endemic country might inform better practices in newly threatened regions. The occurrences of* Echinococcus granulosus* sensu stricto infection among Polish patients are studied and analyzed as the important molecular epidemiology question of whether these result from autochthonous transmission or importation from other countries. Emerging threats for human health in Poland due to drug-resistant* Acanthamoeba* keratitis are also studied, among others in terms of adaptive capability of pathogenic strains, causative agents of the serious, vision-threatening disease.

This general theme of international dissemination of infectious diseases is also examined and analyzed in the review; it relates a wide range of viral, bacterial, and parasitic disease threats to the immigration of adoptees and refugees into the United States from countries around the world. Besides their medical and humanitarian importance, both of these raise significant issues regarding national health policies on both domestic and international issues within the respective countries.

In conclusion, it is important to encourage scientists in extension of our knowledge of serious but poorly recognized national/regional health threats, generated by parasites and related pathogens, intensifying during the last decade. It may allow a new appreciation of the importance of the holistic approach that involves* in vivo* and* in vitro* diagnostic techniques and biomolecular detection including advances in control and prevention strategies and therapeutic procedures as well as practical implications for diagnostics, treatments, and prophylaxis for such parasitic infections and diseases.

*Lidia Chomicz* *David Bruce Conn* *Jacek P. Szaflik* *Beata Szostakowska*
